# Hematopoietic Cell Transplantation for Chronic Granulomatous Disease in Japan

**DOI:** 10.3389/fimmu.2020.01617

**Published:** 2020-07-29

**Authors:** Masakatsu Yanagimachi, Koji Kato, Akihiro Iguchi, Koji Sasaki, Chikako Kiyotani, Katsuyoshi Koh, Takashi Koike, Hideki Sano, Tomonari Shigemura, Hideki Muramatsu, Keiko Okada, Masami Inoue, Ken Tabuchi, Toyoki Nishimura, Tomoyuki Mizukami, Hiroyuki Nunoi, Kohsuke Imai, Masao Kobayashi, Tomohiro Morio

**Affiliations:** ^1^Department of Pediatrics, Tokyo Medical and Dental University, Tokyo, Japan; ^2^Department of Hematology/Oncology, Kanagawa Children's Medical Center, Yokohama, Japan; ^3^Department of Hematology and Oncology, Children's Medical Center, Japanese Red Cross Nagoya First Hospital, Nagoya, Japan; ^4^Central Japan Cord Blood Bank, Seto, Japan; ^5^Department of Pediatrics, Hokkaido University Hospital, Sapporo, Japan; ^6^Department of Pediatrics, Yokohama City University, Yokohama, Japan; ^7^Children's Cancer Center, National Center for Child Health and Development, Tokyo, Japan; ^8^Department of Hematology/Oncology, Saitama Children's Medical Center, Saitama, Japan; ^9^Department of Pediatrics, Tokai University School of Medicine, Isehara, Japan; ^10^Department of Pediatric Oncology, Fukushima Medical University Hospital, Fukushima, Japan; ^11^Department of Pediatrics, Shinshu University School of Medicine, Nagano, Japan; ^12^Department of Pediatrics, Nagoya University Graduate School of Medicine, Nagoya, Japan; ^13^Department of Pediatric Hematology/Oncology, Osaka City General Hospital, Osaka, Japan; ^14^Department of Pediatric Hematology/Oncology, Osaka Women's and Children's Hospital, Osaka, Japan; ^15^Division of Pediatrics, Tokyo Metropolitan Cancer and Infectious Disease Center Komagome Hospital, Tokyo, Japan; ^16^Division of Pediatrics, Developmental and Urological-Reproductive Medicine Faculty of Medicine, University of Miyazaki, Miyazaki, Japan; ^17^Department of Pediatrics, NHO Kumamoto Medical Center, Kumamoto, Japan; ^18^Department of Pediatrics, Hiroshima University Graduate School of Biomedical & Health Sciences, Hiroshima, Japan

**Keywords:** hematopoietic cell transplantation, chronic granulomatous disease, CYBB, adult, cord blood transplantation, low-dose irradiation

## Abstract

Hematopoietic cell transplantation (HCT) is established as a curative treatment for severe chronic granulomatous disease (CGD). However, outcomes of HCT for CGD in Japan had not been precisely reported. We evaluated the outcome of HCT for CGD in Japan by means of a nationwide survey. A total of 91 patients (86 males and 5 females) with CGD who received HCT between 1992 and 2013 was investigated. Their median age at HCT was 11 years (0–39). Sixty-four patients had X-linked CGD caused by CYBB gene mutations, 13 had autosomal recessive CGD (7 CYBA and 6 NCF2), and 14 were genetically undetermined. Seventy patients are still alive at a median follow-up of 38.9 (3.7–230) months. Three-year OS and EFS was 73.7 and 67.6%, respectively. Twenty-one patients died mainly from transplant-related mortality. The cumulative incidence of grade II to IV acute GVHD and extensive chronic GVHD was 27.2 and 17.9%, respectively. Risk factors for EFS after HCT for CGD were age >30 years (*P* < 0.01), non-CYBB gene mutations (*P* < 0.01) and CBT (*P* < 0.01). Regarding the reduced intensity conditioning (RIC) regimen, risk factors for EFS included anti-thymocyte globulin (*P* = 0.048) and not using low-dose irradiation therapy (*P* < 0.01), in addition to the preceding risk factors. We report outcomes of HCT for CGD in Japan. Future studies are needed to improve such outcomes, especially for patients harboring non-CYBB gene mutations and suffering from adult CGD. A RIC regimen including low-dose irradiation may be a good option to explore further.

## Introduction

Chronic granulomatous disease (CGD), caused by a dysfunction of the phagocyte nicotinamide adenine dinucleotide phosphate (NADPH) oxidase system, is an inborn error of immunity (1, 2). CGD is characterized by recurrent and severe infections with bacterial and fungal pathogens and by inflammatory complications, such as granuloma and colitis. Although the natural history of CGD patients has improved because of advances in supportive care, such as anti-fungal prophylaxis, CGD patients still suffer from severe uncontrollable infections or inflammatory complications. Hematopoietic cell transplantation (HCT) is an established therapy for the curative treatment of inborn errors of immunity including severe CGD (3). Recently, improved outcomes of HCT for CGD were reported from other countries (4, 5). However, the outcome of HCT for CGD in Japan had not been precisely reported until now. There are differences in the distribution of the types of molecular defects associated with CGD between Japan and other countries, which may influence the outcome of HCT for CGD (6). With this in mind, we evaluated the outcome of HCT for CGD in Japan by means of a nationwide survey.

## Patients and Methods

### Patients

A total of 91 patients (86 males and 5 females) with CGD who received HCT in 37 HCT centers from 1992 to 2013 was included in this study. They received a total of 100 transplants (2 patients twice, 2 thrice and one patient four times). Analyses in this study were limited to first-time transplants. Median patient age at CGD diagnosis was 0 years (0–19) and median age at first HCT was 11 years (0–39) (Table 1). Sixty-four patients had X-linked CGD caused by CYBB gene mutations, 13 had autosomal recessive (AR) CGD (7 CYBA and 6 NCF2), and 14 were genetically undetermined. The 14 patients were diagnosed CGD only according to their clinical symptoms and low or absent NAPDH-oxidase activity, especially before 2000 or could not be genetically categorized even after genetic analysis. No CGD patient carrying NCF1 and NCF4 gene mutations was identified in this study.

**Table 1:** Patients' characteristics.

**Patients**	**r-HCT (*n* = 38)**	**u-HCT (*n* = 53)**	**Total (*n* = 91)**
Gender (M/F)	36/2	50/3	86/5
Age at diagnosis, median	0.5 (0–19)	0 (0–15)	0 (0–19)
Age at HSCT, median	12.0 (2–35)	9.0 (0–39)	11.0 (0–39)
Genetic mutation			
CYBB	28	36	64
CYBA	2	5	7
NCF2	1	5	6
undetermined	7	7	14
Stem cell source			
HLA-matched BM	23	35	58
HLA-matched PBSC	3	0	3
HLA-mismatched PBSC	2	0	2
HLA-mismatched BM	6	9	15
Haploidentical BM	3	0	3
Cord blood	3^*^	9	12
Conditioning regimen			
MAC	13	11	24
RIC	25	42	67

*r-HCT, related hematopoietic cell transplantation; u-HCT, unrelated hematopoietic cell transplantation; BM, bone marrow; PBSC, peripheral blood stem cell; MAC, myeloablative conditioning; RIC, reduced-intensity conditioning*.

* *One with cord blood and BM, the other with cord blood and PBSC from the same donor, respectively*.

Clinical data were collected by the Japan Society for Hematopoietic Cell Transplantation (JSHCT) and the Japanese Data Center for Hematopoietic Cell Transplantation (JDCHCT) using the Transplant Registry Unified Management Program (TRUMP). Genetic data were collated in the Primary Immunodeficiency database in Japan (PIDJ). This study was approved by the Institutional Review Boards at JSHCT and Tokyo Medical and Dental University. All patients and/or their guardians gave written consent to the JDCHCT and PIDJ.

### Transplantation

The degree of HLA compatibility between donor and recipient was established by serotyping of HLA-A, B, and DR. Twenty-seven patients received a transplant from an HLA-matched sibling donor, of which 23 were bone marrow transplants (BMT), 3 were peripheral blood stem cell transplants (PBSCT), and 3 were cord blood transplants (CBT). Two of the CBT patients received stem cells from two sources simultaneously, namely PBSCT plus CBT, and BMT plus CBT, from HLA-matched sibling donors (Figure S1, Table 1). For statistical analyses, these two patients were assigned to the PBSCT group and BMT group, respectively. Of the patients without a matched sibling donor, 3 received a BMT from haploidentical related donors, and 8 from HLA-mismatched related donors (6 BMT and 2 PBSCT). A total of 53 patients received a transplant from an unrelated donor, including 35 HLA-matched BMT, 9 HLA-mismatched BMT and 9 CBT (1 HLA-8/8 match and 8 HLA-mismatched) (Table 1).

The choice of conditioning regimen was at the discretion of each HCT institution. Myeloablative conditioning (MAC) was defined as a regimen including total body irradiation (TBI), thoraco-abdominal irradiation (TLI) (≥8 Gy, fractionated) or busulfan (BU) (≥8 mg/kg); the other regimens were defined as reduced-intensity conditioning (RIC) [Table 1; (7)]. Six TBI/TLI and 18 BU-based regimens were included in the MAC group, and 27 fludarabine (FLU)/cyclophosphamide (CY), 14 FLU/melphalan (MEL), 19 FLU/CY/MEL and 5 FLU/BU-based regimens were included in the RIC group. For the latter, 48 low-dose TBI (mean 3.3 Gy), 3 TLI (mean 6 Gy), and 4 total abdominal irradiation (TAI) (mean 4.2 Gy) were included, as well as 39 anti-thymocyte globulin (ATG) and 4 anti-lymphocyte globulin (ALG)-treated patients. GVHD prophylaxis was almost always with cyclosporine (*n* = 28) or tacrolimus (*n* = 63) accompanied by methotrexate.

Donor chimerism was defined as >80% donor cells present in the whole white blood cell sample analyzed, whereas mixed chimerism was defined as 20–80% donor chimerism, and graft failure (GF) as <20% donor chimerism, according to the TRUMP classification. Methods of measuring chimerism status were vary, including short tandem repeat analysis and FISH analysis of X/Y chromosome.

Graft versus host disease (GVHD) was scored according to standard criteria (8, 9). The hematopoietic cell transplantation comorbidity index (HCT-CI) was also scored by standard criteria (10).

### Statistical Analysis

Overall survival (OS), event-free survival (EFS), and GVHD-free, event-free survival (GEFS) were described by Kaplan-Meier estimates. Differences among clinical groups were compared using the log-rank test. An event of EFS was defined as GF or death. An event of GEFS was defined as the first event among grades III to IV acute GVHD, extended chronic GVHD, GF, and death. The probability of neutrophil engraftment (≥500/mm^3^), acute and chronic GVHD was evaluated by Gray's test for comparing cumulative incidence curves. For neutrophil engraftment, death before neutrophil recovery was counted as a competing event; for GVHD, death without GVHD was counted as a competing event. The influence of different factors on OS/EFS was evaluated using Cox proportional hazard regression modeling. The influence of different factors on the cumulative incidence of engraftment and GVHD was evaluated using Fine-Gray proportional hazard regression modeling. *P* < 0.05 were considered statistically significant. All analyses were performed with EZR (Saitama Medical Center, Jichi Medical University, Saitama, Japan), which is a graphical user interface for R (the R foundation for statistical Computing, Vienna, Austria) (11).

## Results

### Engraftment and Death

The cumulative incidence of neutrophil engraftment at day 100 was 80.9% for all patients, which broke down to 86.1% in BMT/PBSCT and 40.0% in CBT (Figure S2). Thus, CBT was associated with less neutrophil engraftment, but this was not quite statistically significant (*P* = 0.057). Thirteen GF occurred, after which 10 patients received second or more transplants and 7 survived. GF at day 100 after HCT was associated with poor OS, compared to donor or mixed chimerism (*P* < 0.01; Figure S3). There were two (17%) GF occurred in 13 MAC regimens, 3 (8%) in 37 FLU/CY-based regimens and 5 (31%) in 16 non-FLU/CY-based regimens at day 100 after HCT. In the aspect of stem cell sources, there were 4 (7%) GF occurred in 55 BMT and 6 (67%) in 9 CBT at day 100 after HCT.

In total, 21 patients died, 4 from graft rejection, 4 from GVHD, 2 thrombotic microangiopathy (TMA), 2 infections, 3 hemorrhages, 1 secondary malignancy (osteosarcoma), and 5 others (Table 2).

**Table 2:** Summary of deceased cases.

**No**.	**Gender**	**Age at diagnosis**	**Age at HSCT**	**Genotype**	**Source**	**Conditioning**	**PS**	**HCT-CI**	**Date of death (days)**	**Cause of death**
CGD001	M	0	8	CYBB	HLA-mismatch-u-CBT	MAC	0	0	28	Hemorrhage
CGD002	M	0	5	CYBB	HLA-match-r-PBSCT and CBT	MAC	0	0	77	Acute GVHD
CGD003	M	0	5	CYBB	HLA-match-r-CBT	MAC	0	0	22	Multiple organ failure
CGD004	F	0	3	CYBA	HLA-match-u-BMT	RIC	3	0	28	Rejection
CGD007	M	0	4	CYBB	HLA-mismatch-r-BMT	MAC	0	0	20	Rejection
CGD009	M	1	12	CYBB	HLA-mismatch-r-PBSCT	RIC	0	0	28	Hemorrhage
CGD013	M	0	11	CYBB	HLA-match-r-BMT	MAC	0	0	156	Infection
CGD015	F	10	31	CYBA	HLA-mismatch-r-BMT	MAC	1	2	83	Acute GVHD
CGD017	M	7	8	CYBB	HLA-mismatch-r-BMT	MAC	0	0	2,441	Secondary malignancy
CGD018	M	0	17	N.D.	HLA-mismatch-u-BMT	RIC	0	0	54	Hemorrhage
CGD020	F	1	22	CYBA	HLA-match-u-BMT	MAC	0	0	22	Multiple organ failure
CGD022	M	0	12	N.D.	HLA-match-u-BMT	RIC	0	0	1,210	Chronic GVHD
CGD024	M	0	18	CYBB	HLA-match-r-BMT	RIC	3	4	780	Chronic GVHD
CGD025	M	0	30	CYBB	HLA-match-u-BMT	RIC	1	0	217	Multiple organ failure
CGD026	M	0	9	CYBA	HLA-mismatch-u-BMT	RIC	0	0	100	ARDS
CGD029	M	0	35	CYBB	HLA-match-r-BMT	RIC	1	5	319	TMA
CGD038	M	0	3	CYBB	HLA-mismatch-u-BMT	RIC	0	0	46	Infection
CGD039	M	0	1	NCF2	HLA-mismatch-u-CBT	MAC	2	4	28	Rejection
CGD040	M	7	15	NCF2	HLA-mismatch-u-CBT	RIC	1	4	28	Rejection
CGD046	M	3	39	CYBB	HLA-match-u-BMT	RIC	0	0	425	TMA
CGD047	M	2	3	NCF2	HLA-match-u-BMT	RIC	0	0	49	Cerebral infarction

*CBT, cord blood transplantation; BMT, bone marrow transplantation; MAC, myeloablative conditioning; RIC, reduced-intensity conditioning; PS, performance status; HCT-CI, hematopoietic cell transplantation comorbidity index; GVHD, graft versus host disease; ARDS, acute respiratory distress syndrome; TMA, thrombotic microangiopathy; N.D, not determined*.

### GVHD

The cumulative incidence of grade II to IV acute GVHD was 25.3% and the incidence of extensive chronic GVHD was 19.6%. ATG/ALG, donor source and HLA compatibility were not associated with the incidence of grade II to IV acute GVHD and extensive chronic GVHD.

### EFS/OS and GEFS

**Figure 1:**
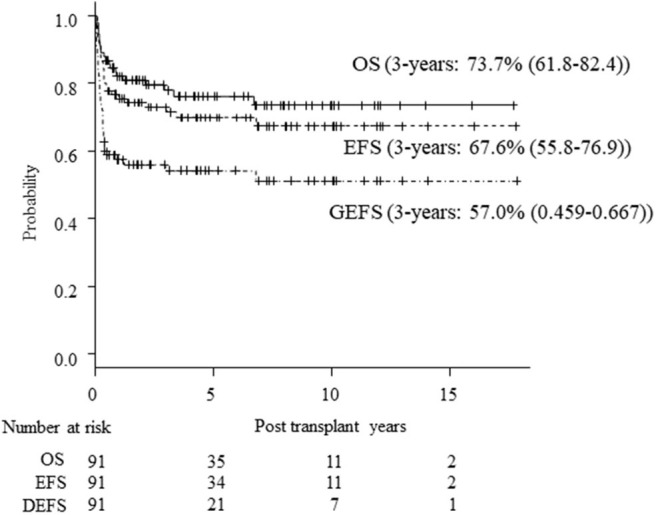
Kaplan-Meier estimates of OS/EFS/GEFS in all patients. Kaplan-Meier estimates of overall survival (OS), event-free survival (EFS), and GVHD-free, event-free survival (GEFS) of CGD patients undergoing HCT.

**Figure 2:**
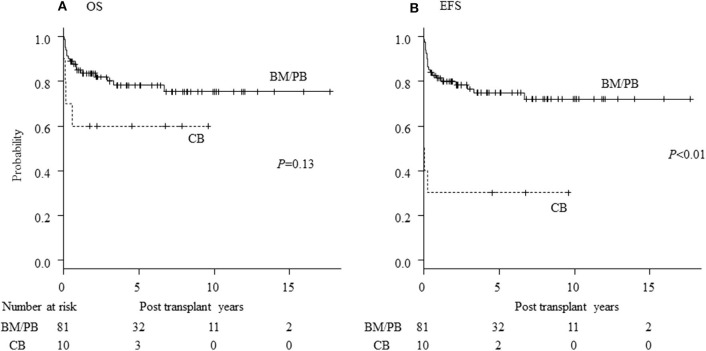
Influence of stem cell source for HCT of CGD. Influence of stem cell source of transplantation on **(A)** overall survival (OS) and **(B)** event-free survival (EFS) of CGD patients undergoing HCT. CB, cord blood cell transplantation; BM, bone marrow transplantation; PB, peripheral blood stem cell transplantation.

Seventy patients are alive at a median follow-up of 38.9 (3.7–230) months. Three-year OS, EFS and GEFS was 73.7, 67.6, and 57.0%, respectively (Figure 1). Twenty-one patients died mainly from transplant-related mortality (TRM) (Table 2). Eighteen male and 3 female patients died (*P* = 0.08). CBT was a significant risk factor for low EFS/GEFS (*P* < 0.01), but not for OS (*P* = 0.12; Figure 2, Table 3).

**Table 3:** Outcome of CBT for CGD.

**No**.	**Gender**	**Age at diagnosis**	**Age at HSCT**	**Genotype**	**Donor**	**HLA**	**Conditioning**	**PS**	**HCT-CI**	**Graft failure/chimerism**	**Result**
CGD001	M	0	8	CYBB	Unrelated	7/8	MAC	0	0	GF	Dead
CGD003	M	0	5	CYBB	Sibling	6/6	MAC	0	0	GF	Dead
CGD039	M	0	1	NCF2	Unrelated	5/6	MAC	2	4	GF	Dead
CGD040	M	7	15	NCF2	Unrelated	5/8	RIC	1	4	GF	Dead after 2nd HSCT
CGD051	M	0	0	CYBB	Unrelated	6/6	RIC	1	2	Mixed chimera	Alive after 2nd HSCT
CGD073	F	0	0	NCF2	Unrelated	4/6	RIC	3	1	GF	Alive after 2nd HSCT
CGD084	M	0	7	CYBB	Unrelated	5/6	MAC	1	1	Donor chimera	Alive
CGD091	M	4	5	CYBB	Unrelated	6/8	RIC	0	1	GF	Alive after 2nd HSCT
CGD093	M	1	20	CYBB	Unrelated	4/8	RIC	0	0	Donor chimera	Alive
CGD101	M	0	21	CYBB	Unrelated	3/6	RIC	0	0	GF	Alive waiting for HSCT

*CBT, cord blood transplantation; MAC, myeloablative conditioning; RIC, reduced-intensity conditioning; PS, performance status; HCT-CI, Hematopoietic cell transplantation comorbidity index; GF, graft failure*.

**Figure 3:**
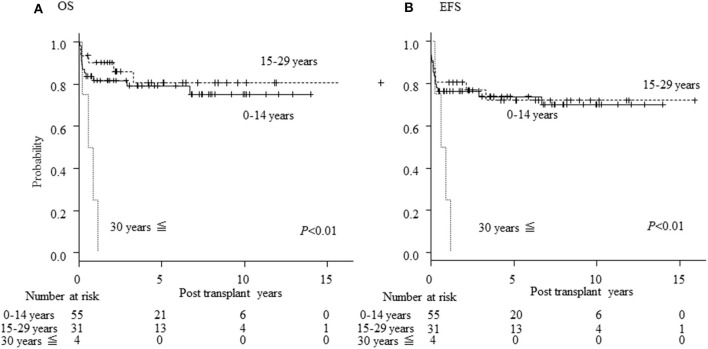
Influence of age on outcome of HCT. Influence of age at HCT on outcome. **(A)** Overall survival; **(B)** event-free survival of CGD patients undergoing HCT.

There was no difference in OS/EFS/GEFS(OK) between patients transplanted 2008-2013 or earlier either for all patients or in the CBT setting only. Age >30 years at HCT resulted in poorer OS/EFS/GEFS than in younger patients (OS; *P* < 0.01/ EFS; *P* < 0.01/GEFS; *P* = 0.04; Figure 3). A total of 4 patients aged >30 years at HCT died from TRM (Table 2). A high HCT-CI score (over 3) also influenced the outcome of HCT in all patients (Figure S4).

**Figure 4:**
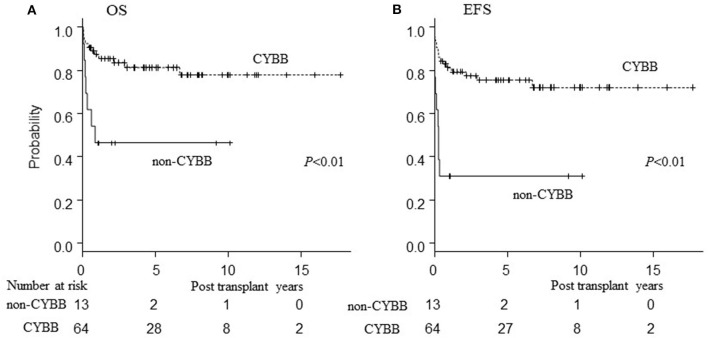
Influence of genetic mutation on outcome of HCT. Influence of genetic mutation on outcome of HCT. **(A)** Overall survival; **(B)** event-free survival of CGD patients undergoing HCT. CYBB, patients with CYBB gene mutation. Non-CYBB, patients with CYBA or NCF2 gene mutations.

Patients harboring CYBA or NCF2 (non-CYBB) gene mutations had poor OS/EFS/GEFS(OK) compared to those with CYBB mutations (*P* < 0.01; Figure 4). There was no difference in the patients' characteristics between those with or without CYBB mutations [Table S1; (10)].

Active bacterial or fungal infection at the time of HCT did not influence OS/EFS/GEFS in this cohort.

For BMT from HLA-matched donors, there was no difference in OS/EFS/GEFS between related (*n* = 27) and unrelated (*n* = 36) donor groups (OS; *P* = 0.67/EFS; *P* = 0.50/GEFS). Also for BMT from unrelated donors, no differences were seen in OS/EFS/GEFS; z between HLA fully matched donors (*n* = 19) and donors mismatched for one allele (*n* = 9) (OS; *P* = 0.64/EFS; *P* = 0.77/GEFS; *P* = 0.73). No differences were seen in OS/EFS/GEFS between HLA fully matched related donors and the other donors in all patients (OS; *P* = 0.58/EFS; *P* = 0.18/GEFS; *P* = 0.61) and in patients received HCT with RIC regimens (OS; *P* = 0.30/EFS; *P* = 0.07/GEFS; *P* = 0.10).

### Conditioning Regimens

A total of 24 patients on different MAC regimens and 67 on RIC regimens had been selected at the discretion of each institution (Table 1). After the year 2000, mainly RIC regimens were employed. There were no differences in OS/EFS between patients receiving RIC or MAC regimens by univariate analysis (Figure S5). Thus, in the univariate analysis, ATG/ALG and TBI/TLI/TAI use was not associated with OS/EFS in the whole patient cohort or the RIC group (Figure S6). EFS was higher in the 47 patients receiving FLU/CY-based regimens (*n* = 27) and FLU/CY/MEL regimens (*n* = 19) than in the 19 patients not receiving FLU/CY-based regimens [FLU/BU (*n* = 5) and FLU/MEL (*n* = 14)] (*p* = 0.02) in the RIC group (Figure S7).

By multivariate analysis in the Cox proportional hazard regression model, risk factors for EFS of HCT for CGD in all patients were found to be age >30 years (*P* < 0.01), CBT (*P* < 0.01) and non-CYBB mutation (*P* < 0.01). Age >30 years (*P* < 0.01), MAC (*P* = 0.03) and non-CYBB mutation (*P* = 0.03) were also risk factors for OS in all patients (Table 4). Although there was no risk factor for GEFS in all patients, CBT tended to be associated with lower GEFS (*P* = 0.068). Risk factors for EFS of HCT for CGD in patients receiving RIC regimens were again age >30 years (*P* < 0.01), CBT (*P* < 0.01), non-CYBB mutation (*P* < 0.01), and also ATG/ALG (*P* = 0.048) and non-TBI/TLI/TAI (*P* < 0.01). Age over 30 years (*P* < 0.01), non-CYBB mutation (*P* < 0.01) and HCT-CI score (over 3) (*P* = 0.02) were also risk factors for OS in patients on RIC regimens (Table 4).

**Table 4:** Cox regression analysis for risk factors.

**Factor**	**Hazard ratio**	***p*-value**	**Factor**	**Hazard ratio**	***p*-value**
**OS for all patients (*****n*** **=** **91)**			**EFS for all patients (*****n*** **=** **91)**		
Age (over 30 years)	6.48 (1.86–22.6)	<0.01	Age (over 30 years)	4.89 (1.52–15.7)	<0.01
Cord blood transplantation	1.81 (0.49–6.67)	0.34	Cord blood transplantation	6.01 (2.23–16.2)	<0.01
CYBB	0.32 (0.11–0.89)	0.03	CYBB	0.21 (0.09–0.52)	<0.01
RIC	0.35 (0.13–0.92)	0.03	RIC	0.75 (0.32–1.76)	0.51
HCT-CI score (over 3)	2.68 (0.76–9.48)	0.13	HCT-CI score (over 3)	1.77 (0.55–5.74)	0.34
**OS for RIC regimens (*****n*** **=** **67)**			**EFS for RIC regimens (*****n*** **=** **67)**		
Age (over 30 years)	169 (8.54–3367)	<0.01	Age (over 30 years)	21.8 (3.93–121.2)	<0.01
Cord blood transplantation	0.06 (0.00–1.79)	0.79	Cord blood transplantation	7.76 (1.69–35.6)	<0.01
CYBB	0.01 (0.00–0.19)	<0.01	CYBB	0.07 (0.02–0.28)	<0.01
HCT-CI score (over 3)	17.5 (1.64–187)	0.02	HCT-CI score (over 3)	1.99 (0.49–8.03)	0.34
FLU/CY-based	0.25 (0.05–1.26)	0.09	FLU/CY-based	0.83 (0.26–2.62)	0.75
Low-dose irradiation	0.12 (0.01–1.23)	0.07	low-dose irradiation	0.13 (0.03–0.57)	<0.01
ATG/ALG	9.56 (0.94–97.3)	0.06	ATG/ALG	3.69 (1.01–13.4)	0.048

*RIC, reduced-intensity conditioning; HCT-CI, The hematopoietic cell transplantation comorbidity index; FLU/CY, “fludarabine and cyclophosphamide with/without melphalan” regimens; low-dose irradiation, total body irradiation (TBI), thoraco-abdominal irradiation (TLI), and total abdominal irradiation (TAI). ATG, anti-thymocyte globulin; ALG, anti-lymphocyte globulin*.

## Discussion

HCT is an established curative treatment for severe CGD (4, 5, 12). However, overall results of HCT for CGD in Japan had not been precisely reported until now. Therefore, we carried out a nationwide survey of the outcome of HCT for CGD in Japan. Three-year OS (73.7%) in our cohort is very similar to that reported in previous publications, except for Güngör's study with HLA-matched donors (4, 5, 12–15). Recently, improved outcomes of HCT for CGD were reported from other countries (4, 5). HCT with “submyeloablative” conditioning regimens using low-dose or targeted busulfan administration for CGD resulted in excellent 2-year EFS (91%) and stable donor chimerism (93%) (4). Treosulfan-based conditioning resulted in 81% EFS (median follow-up, 34 months) without severe adverse events such as sinusoidal obstruction syndrome (5). These procedures will be introduced for Japanese CGD patients shortly.

This present report is the first to document poor HCT outcomes for CGD patients harboring non-CYBB gene mutations, whereas previous studies had noted that non-CYBB gene mutations were associated with better HCT outcome than CYBB mutations (4, 5, 15). In general, CGD patients harboring non-CYBB gene mutations have less severe infections and lower mortality rates than with CYBB mutations (16). Indications for HCT, especially for CGD patients with non-CYBB gene mutations, have not been established. However, patients harboring non-CYBB mutations in our cohort tended to have high HCT-CI scores (Table S1). Although there was no data of NAPDH-oxidase activity of these CGD patients harboring non-CYBB gene mutations, they might have low or absent NAPDH-oxidase activity that caused severe phenotype of these patients. These severe general conditions before and at the time of HCT might have been an influence contributing to the poor outcome of non-CYBB CGD patients in the present study. There are also possible differences in the outcomes of HCT for CGD with non-CYBB gene mutations between countries; thus, these mutations are associated with lower incidence of GF and death after HCT in other countries [Table S2; (4, 5, 15, 17, 18)]. It is necessary to evaluate the outcomes of HCT for CGD patients with non-CYBB gene mutations in detail internationally, because of rarity of these gene mutation subtypes of CGD.

Adult CGD patients are a high-risk group for HCT, because of organ dysfunction, overt infections and inflammatory and post-inflammatory complications (14, 19). Although there was no difference in the outcome of HCT between patients grouped according to the age range 0–14 years compared with 15–29 years, all 4 patients >30 years old in this study died from TRM, such as TMA and acute GVHD (Figure 3, Table 2). It was reported that donor cell infusion in CGD patients with GF or poor donor chimerism after HCT was associated with poor outcome (20). In this study, three of the four died after donor lymphoid infusion, which should therefore be used with great caution for treating patients with GF or mixed chimerism.

Mixed chimerism did not influence OS/EFS in this study (Figure S3). Older female carriers of CGD tended to suffer more complications, such as suppurative infections, gastrointestinal manifestations, and autoimmunity that CGD patients frequently experience (21, 22). Long term follow-up of CGD patients with mixed chimerism after HCT is needed for evaluating whether these complications may occur at a later time in such patients.

CBT was correlated with GF and death in this study, consistent with a previous report [Figure 2, Table 3; (23)]. It was also reported that the outcome of CBT for CGD using MAC was acceptable (24). Recently, the use of “submyeloablative” busulfan-based and treosulfan-based conditioning regimens for BMT and PBSCT has resulted in excellent engraftment and OS. These regimens are expected also to deliver promising results in unrelated CBT for CGD (4, 5). In the present study, we determined that using RIC regimens is associated with better OS than MAC regimens and that low-dose TBI/TLI/TAI resulted in higher EFS rates than RIC without TBI/TLI/TAI (Table 4). Prospective evaluations are now warranted as to whether addition of low-dose irradiation in “submyeloablative” busulfan- or treosulfan- based regimens with HLA-mismatched donor and the CBT setting will improve outcomes of HCT, according to the previous research (25). ATG/ALG was a risk factor for EFS in RIC regimens but was not associated with post-HCT viral and fungal infections (*p* = 0.55).

Here, we report the first nationwide study of HCT for CGD in Japan. However, this is a retrospective study using the TRUMP and PIDJ databases. Therefore, there are intrinsic limitations to the study, such as data quality regarding donor chimerism status, HLA compatibility, and CGD genotyping. The TRUMP database does not fully include CGD-related complications before HCT, such as inflammatory bowel disease. Therefore, we could not adequately evaluate the influence of the patient's condition pre-HCT on the outcome of HCT, although this is probably an important factor. Prospective studies are needed to establish the most effective HCT conditioning regimen for CGD patients. The establishment of eligibility criteria for HCT for severe CGD is also needed. Meanwhile, the outcome of HCT in adult CGD patients under 30 years old was as good as younger patients. It was reported that 3-years OS was 81.8% for adult CGD patients who had HCT [*n* = 11, median age at HCT; 19 (17–27)] (26). When a CGD patient with absent NAPDH-oxidase activity suffers from uncontrollable infections or severe inflammatory complications and has a suitable donor, HCT may be the treatment of choice, especially in patients younger than 30 years old, but if the patient has no available matched donor, gene therapy, or HLA-haploidentical BMT/PBSCT should be considered.

We could not adequately evaluate the late effects of HCT for CGD patients, such as issues with fertility and secondary malignancy. Anecdotally, we can say that some CGD patients who had HCT with certain RIC regimens have in the meantime born children (personal communication). Therefore, long term follow-up will be required to establish the nature of late effects of HCT and for the evaluation of the best HCT conditioning regimen and age at HCT.

We have reported on outcomes of HCT for CGD in Japan. Future studies are needed to improve these outcomes, especially for patients harboring non-CYBB gene mutations or who are adults. Improved outcomes of HCT for CGD were recently reported, and RIC regimens using low-dose irradiation therapy may be one of the best approaches.

## Data Availability Statement

The datasets presented in this article are not readily available because the dataset belongs to the Japan Society for Hematopoietic Cell Transplantation (JSHCT) and the Japanese Data Center for Hematopoietic Cell Transplantation (JDCHCT). Requests to access the datasets should be directed to http://www.jdchct.or.jp/.

## Ethics Statement

The studies involving human participants were reviewed and approved by the Institutional Review Boards at JSHCT and Tokyo Medical and Dental University. Written informed consent to participate in this study was provided by the participants' legal guardian/next of kin.

## Author Contributions

MY designed the research, analyzed the data, and wrote the paper. KKa, AI, and MK analyzed the data and wrote the paper. KS, CK, KKo, TK, HS, HM, and KO performed the research and provided the clinical data. MI and TK contributed in the transplantation data management as the members of Japanese Data Center for Hematopoietic Cell Transplantation and analyzed the data. TN, TMi, HN, and KI analyzed the gene mutation causing CGD. KI and TMo designed the research and wrote the paper. All authors contributed to the article and approved the submitted version.

## Conflict of Interest

The authors declare that the research was conducted in the absence of any commercial or financial relationships that could be construed as a potential conflict of interest.
