# Supplementary material for: Hematopoietic Cell Transplantation for Chronic Granulomatous Disease in Japan
Source: Front Immunol. 2020 Jul 29;11:1617. doi: 10.3389/fimmu.2020.01617 (PMC7403177; doi:10.3389/fimmu.2020.01617)
Supplement: Supplementary file 1 [file Data_Sheet_1.zip › Supplemental Figure Legends.docx]

FIGURE S1. Stem cell source of HCT for CGD in Japan

Circular chart for stem cell source of hematopoietic cell transplantation.

BM, bone marrow transplantation. PB, peripheral blood stem cell transplantation. CB, Cord blood cell transplantation.

FIGURE S2. Cumulative incidence of neutrophil engraftment

Cumulative incidence of neutrophil engraftment of (A) all patients (n=91). (B) Influence of stem cell source on neutrophil engraftment.

BM, bone marrow transplantation. PB, peripheral blood stem cell transplantation. CB, Cord blood cell transplantation.

FIGURE S3. OS classified by day 100 chimerism

Kaplan-Meier estimates of overall survival (OS) of CGD patients undergoing HCT classified by day 100 chimerism.

Donor chimerism was defined as higher than >80% donor cells in whole white blood cells; mixed chimerism as 20-80% donor chimerism; graft failure as<20% donor chimerism.

FIGURE S4. Influence of HCT-CI on outcome of HCT

Influence of HCT-CI on outcome of HCT. (A) overall survival; (B) event-free survival of CGD patients undergoing HCT.

HCT-CI, hematopoietic cell transplantation comorbidity index.

FIGURE S5. Influence of conditioning regimen on outcome

Kaplan-Meier estimates of (A) overall survival (OS); (B) event-free survival (EFS) of CGD patients undergoing HCT after myeloablative (MAC) or reduced-intensity (RIC) conditioning.

FIGURE S6. Influence of low-dose TBI in RIC regimens on outcome

Influence of low-dose TBI/TLI/TAI in RIC regimens on outcome of HCT. (A) overall survival; (B) event-free survival of CGD patients undergoing HCT.

TBI, total body irradiation. TLI, thoraco-abdominal irradiation. TAI, total abdominal irradiation. RIC, reduced-intensity conditioning.

FIGURE S7. Influence of FLU/*CY-*based regimens in RIC regimens on outcome of HCT

Influence of *CY-*based regimens in RIC on the outcome of HCT. (A) overall survival; (B) event-free survival of CGD patients undergoing HCT.

FLU/CY, “fludarabine and cyclophosphamide with/without melphalan” regimens. Non-FLU/CY, “fludarabine and melphalan” or “fludarabine and busulfan” regimens.
