# Supplementary material for: Hematopoietic Cell Transplantation for Chronic Granulomatous Disease in Japan
Source: Front Immunol. 2020 Jul 29;11:1617. doi: 10.3389/fimmu.2020.01617 (PMC7403177; doi:10.3389/fimmu.2020.01617)
Supplement: Supplementary file 1 [file Data_Sheet_1.zip › Supplemental Figures.pdf]

FIGURE S1. Stem cell source of HCT for CGD in Japan

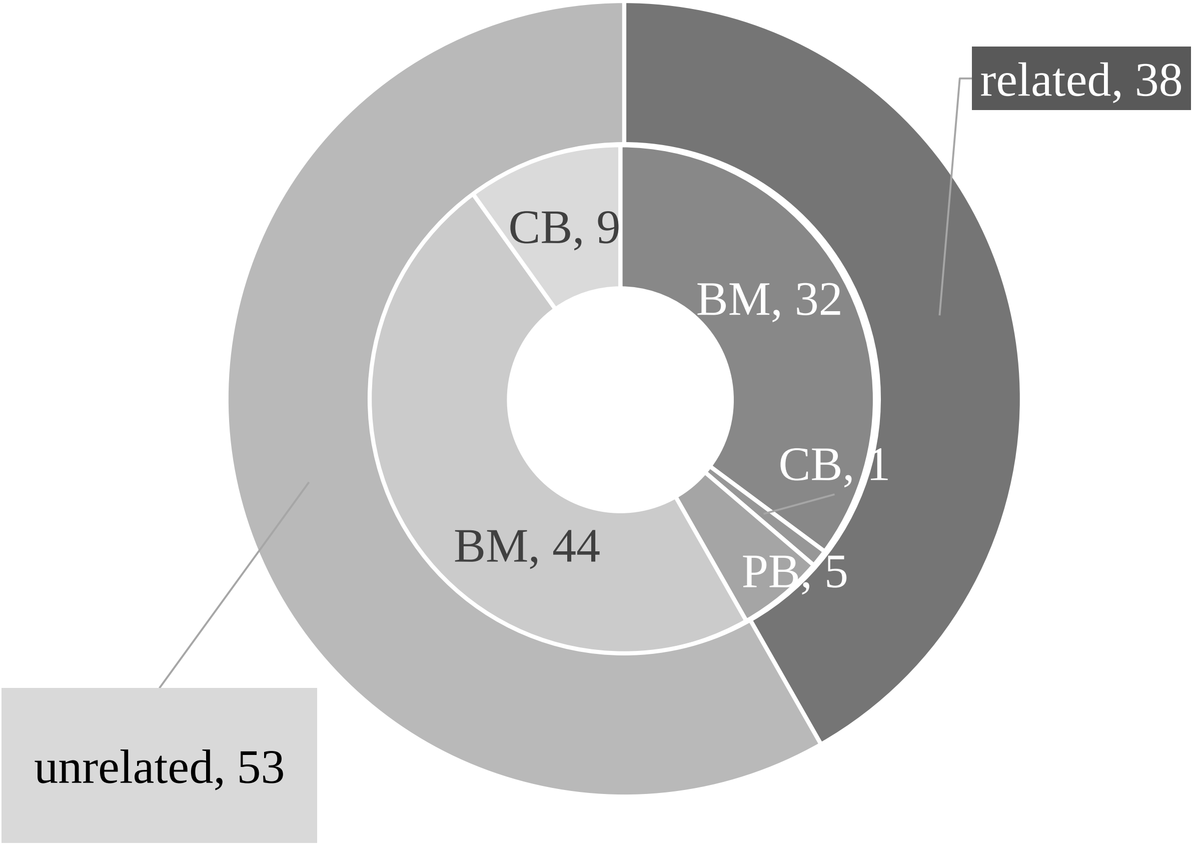

FIGURE S2. Cumulative incidence of neutrophil engraftment

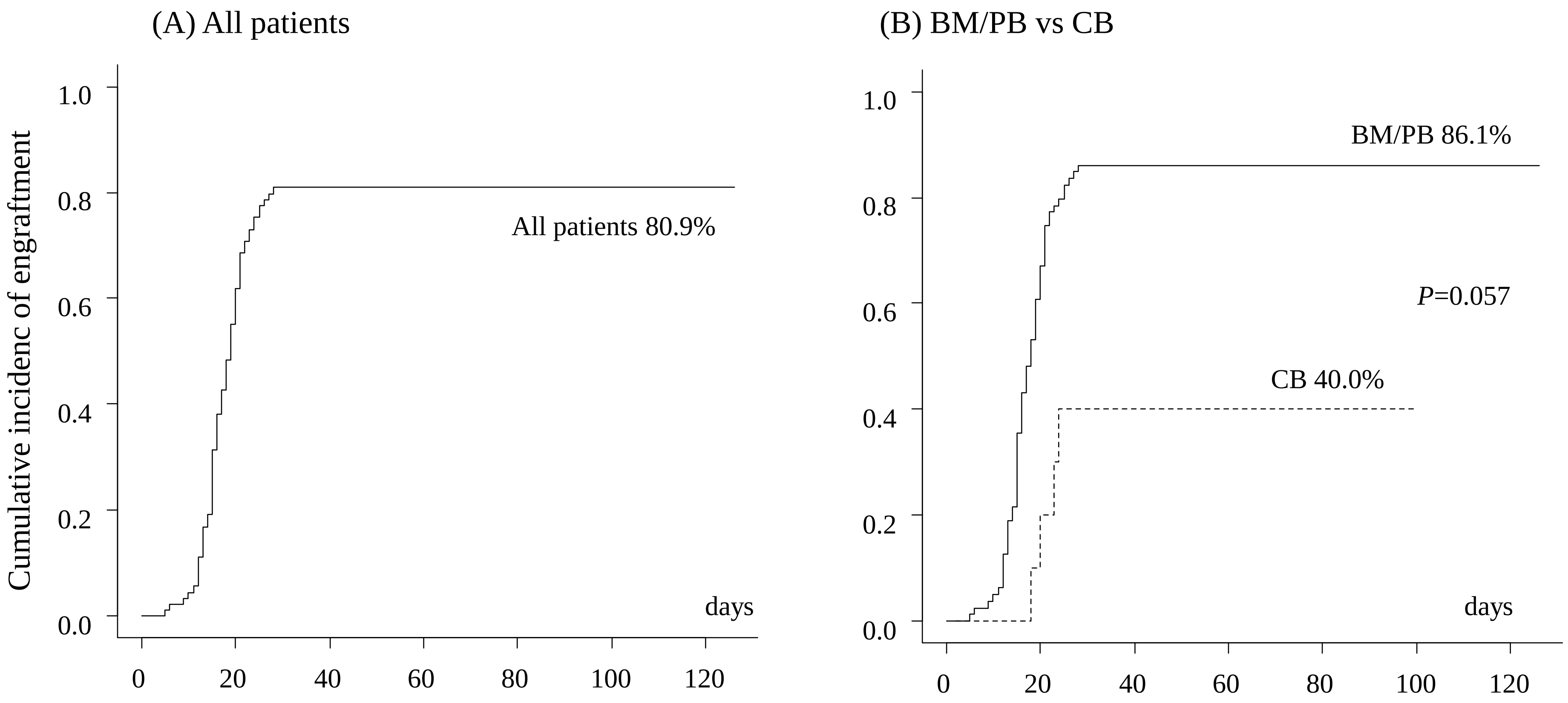

FIGURE S3. OS classified by day 100 chimerism

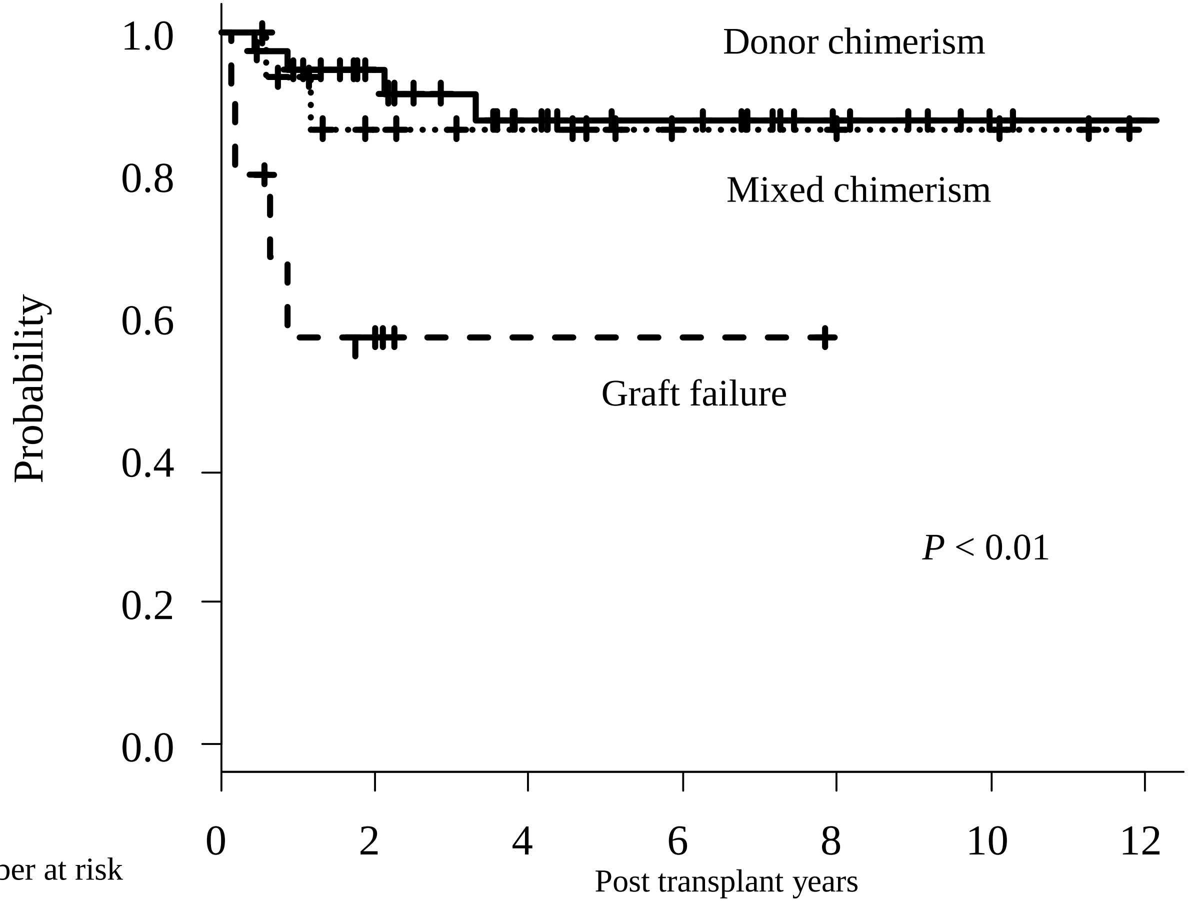

Number at risk

	0	2	4	6	8	10	12
Donor chimerism	39	29	18	14	7	2	1
Mixed chimerism	17	10	8	4	3	3	0
Graft failure	10	4	1	1	0	0	0

FIGURE S4 Influence of HCT-CI on outcome of HCT

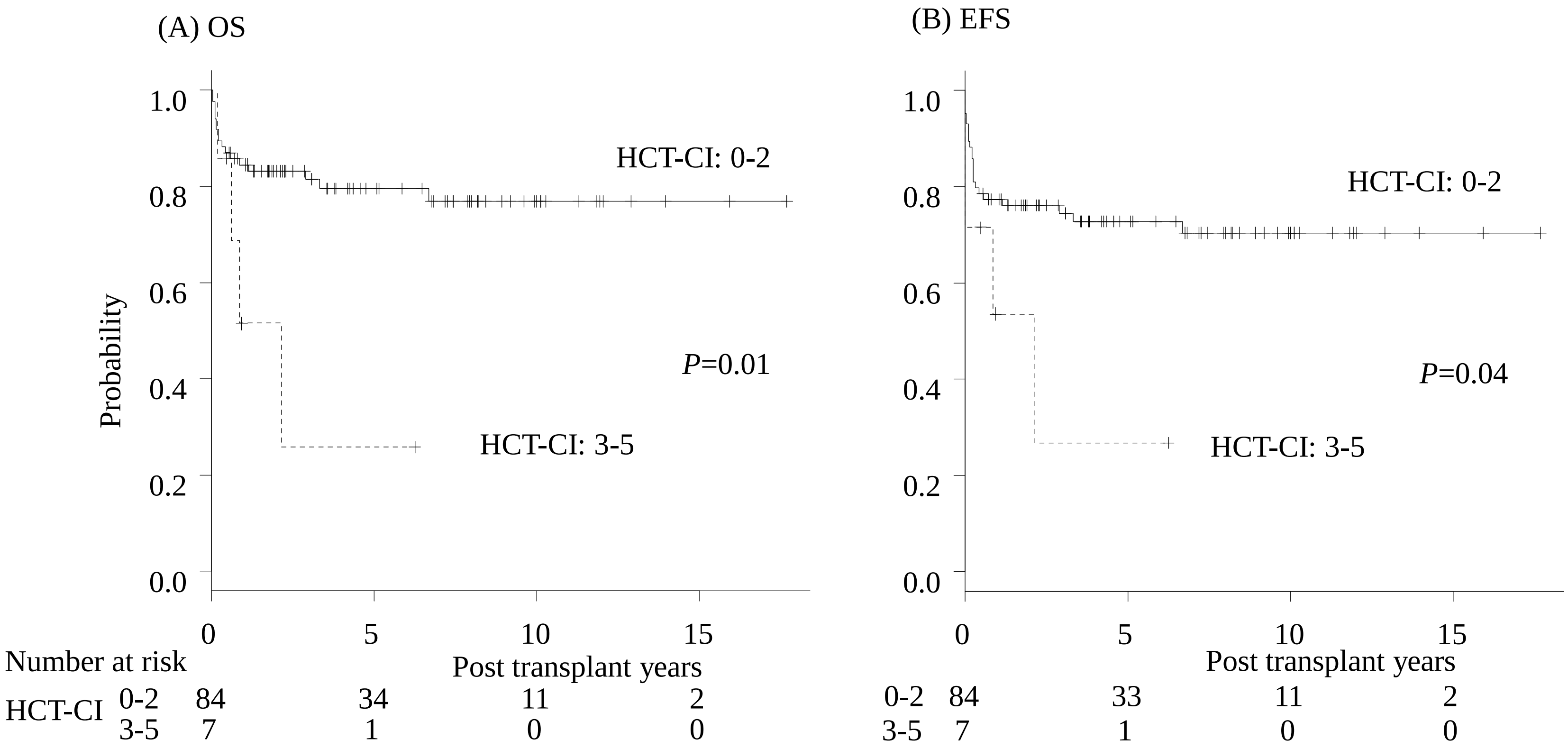

FIGURE S5. Influence of conditioning regimen on outcome

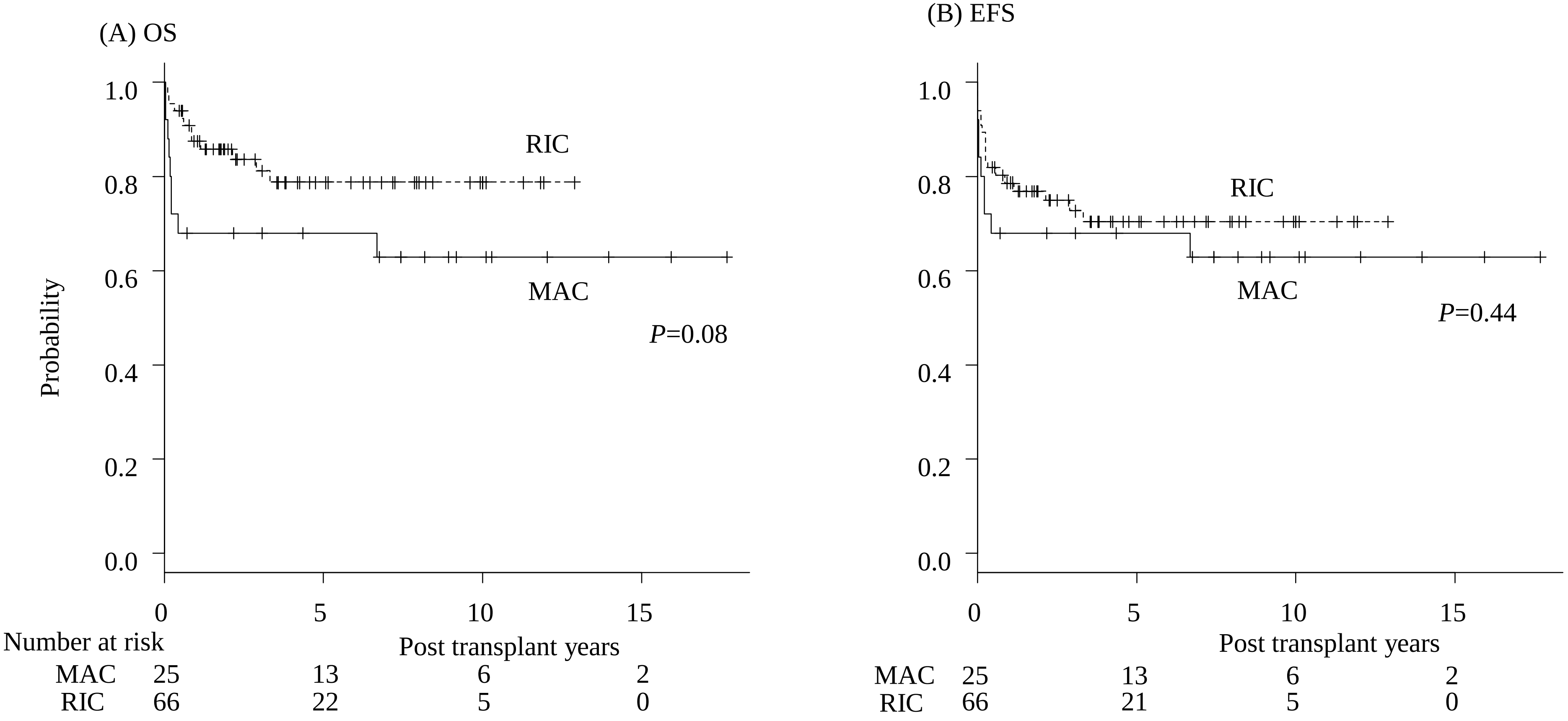

FIGURE S6 Influence of low-dose TBI in RIC regimens on outcome

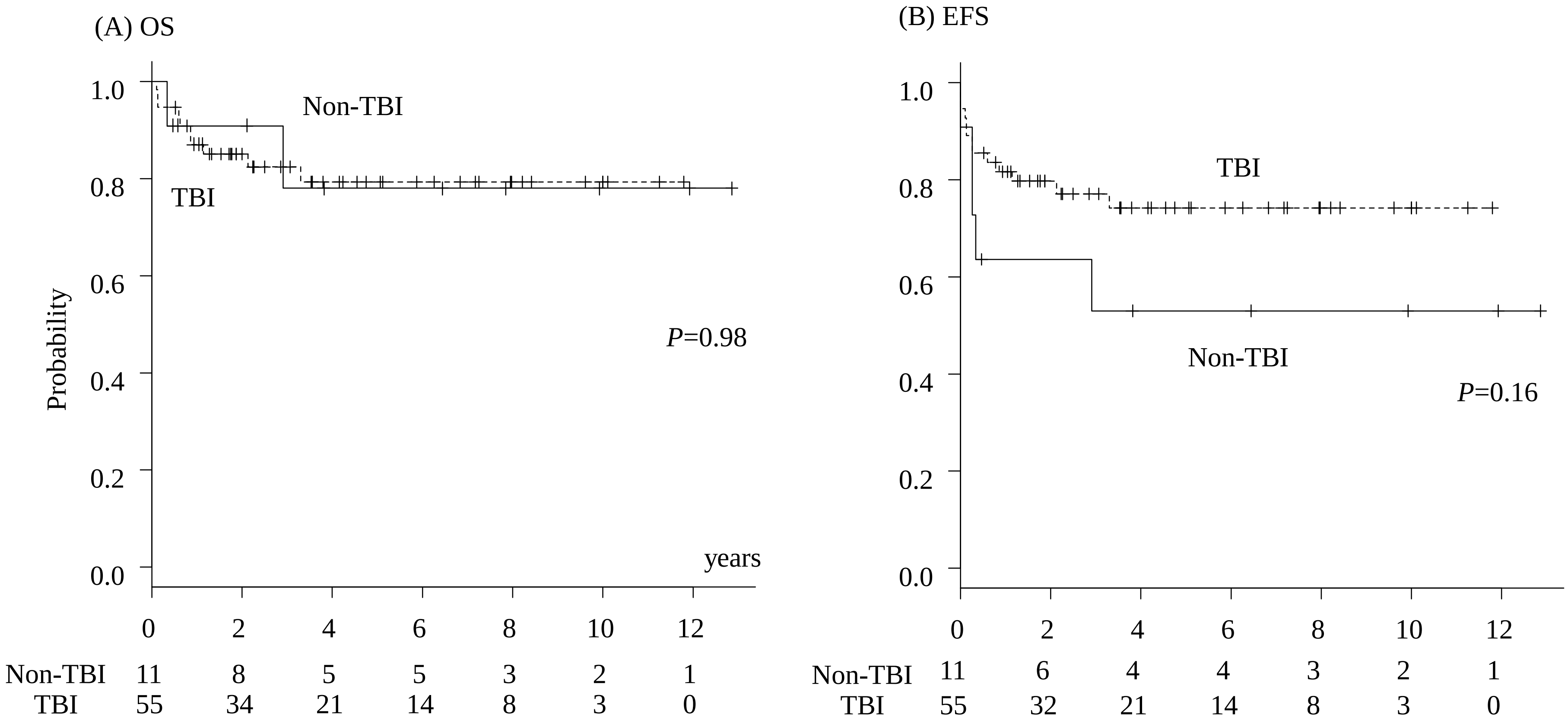

FIGURE S7 Influence of FLU/CY-based regimens in RIC regimens on outcome of HCT

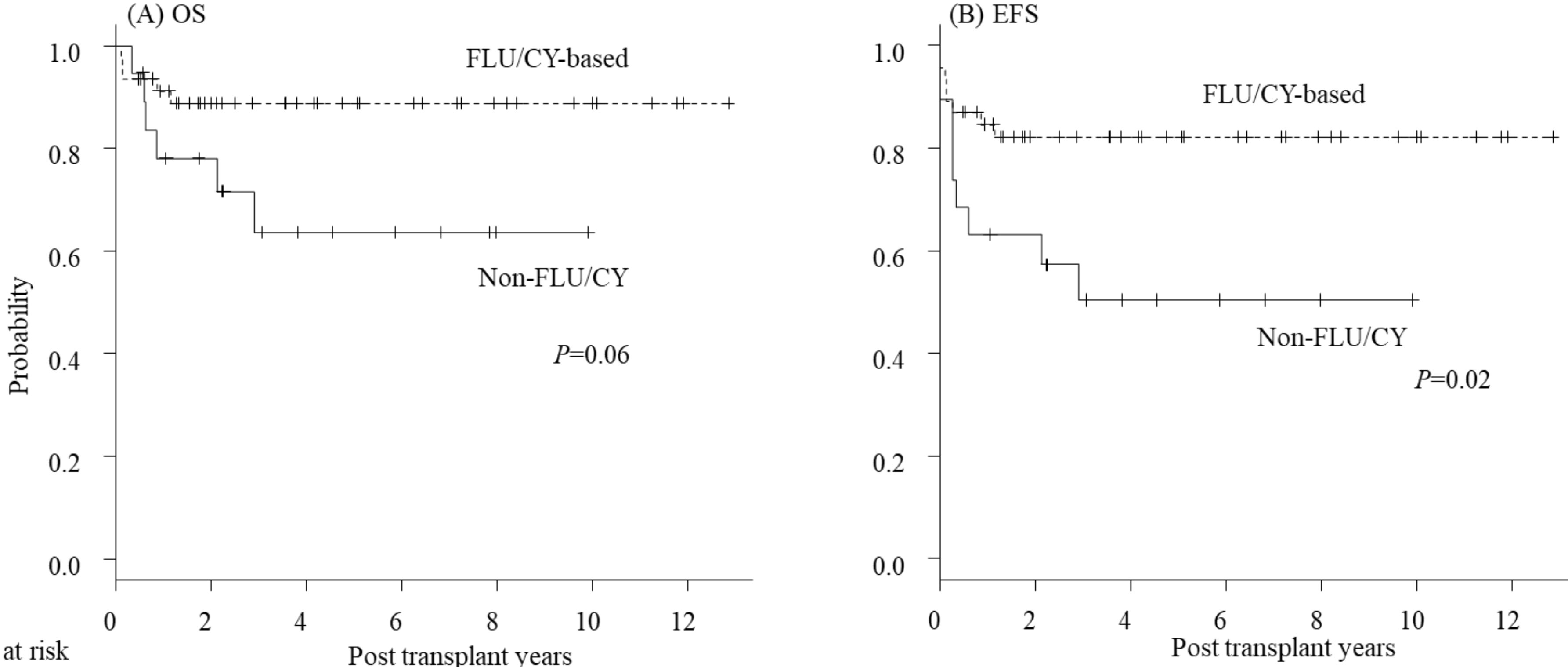

(A) OS

Number at risk							
Non-FLU/CY	19	12	6	4	1	0	0
FLU/CY-based	46	29	20	15	10	5	1

(B) EFS

Non-FLU/CY	19	11	5	3	1	0	0
FLU/CY-based	46	26	20	15	10	5	1
