# Supplementary material for: Hematopoietic Cell Transplantation for Chronic Granulomatous Disease in Japan
Source: Front Immunol. 2020 Jul 29;11:1617. doi: 10.3389/fimmu.2020.01617 (PMC7403177; doi:10.3389/fimmu.2020.01617)
Supplement: Supplementary file 1 [file Data_Sheet_1.zip › Supplemental Tables.docx]

| TABLE S1. Characteristics of patients with different CGD genotypes | | | | |
| --- | --- | --- | --- | --- |
| Genotype |  | CYBA/NCF2 | CYBB | p.value |
| (n) |  | (13) | (64) |  |
| Age at HSCT | year, median (range) | 9 (0-31) | 11 (0-39) | 0.93 |
| Sex | male, n (%) | 8 (61.5) | 64 (100) | <0.01 |
|  | female, n (%) | 5( 38.5) | 0 (0) |  |
| Calendar periods of HSCT | -2007, n (%) | 4 (30.8) | 34 (53.1) | 0.22 |
|  | 2008-2013, n (%) | 9 (69.2) | 30 (46.9) |  |
| Conditioning | MAC, n (%) | 4 (30.8) | 18 (28.1) | 1 |
|  | RIC, n (%) | 9 (69.2) | 46 (71.9) |  |
| Cord blood transplantation | Yes, n (%) | 3 (23.1) | 7 (10.9) | 0.36 |
|  | No, n (%) | 10 (76.9) | 57 ( 89.1) |  |
| HCT-CI | 0-2, n (%) | 11 (84.6) | 59 (92.2) | 0.34 |
|  | 3-5, n (%) | 2 (15.4) | 5 ( 7.8) |  |
| GF or death | Yes, n (%) | 9 (69.2) | 16 (25.0) | <0.01 |
|  | No, n (%) | 4 (30.8) | 48 (75.0) |  |
|  |  |  |  |  |
| GF: graft failure |  |  |  |  |
| HCT-CI: Hematopoietic cell transplantation comorbidity index | | |  |  |
| GVHD: graft versus host disease | |  |  |  |

| TABLE S2. Summary of the influence of CGD genotype on the outcome of HCT | | | | | | | |  |  |  |
| --- | --- | --- | --- | --- | --- | --- | --- | --- | --- | --- |
|  | CYBB | | CYBA | | NCF1 | | NCF2 | | Not determined | |
|  | (gp91-) | | (p22-) | | (p47-) | | (p67-) | |  |  |
| Event (death/graft failure) | + | - | + | - | + | - | + | - | + | - |
| Gungor, et al. 2014(n=56) | 5 | 30 | 0 | 2 | 1 | 9 | 0 | 4 | 0 | 5 |
| Gutierrez, et al. 2016 (n=70) | 11 | 46 | 0 | 4 | 0 | 4 | 0 | 1 | 0 | 4 |
| Seger, et al. 2002 (n=27) | 4 | 19 | 0 | 1 | 0 | 1 | 0 | 0 | 0 | 2 |
| Horwitz, et al.2001 (n=10) | 2 | 6 | 0 | 1 | 0 | 1 | 0 | 0 | 0 | 0 |
| Kutukculer, et al.2019(n=9) | 2 | 3 | 1 | 1 | 0 | 2 | 0 | 0 | 0 | 0 |
| **This study (n=91)** | **19** | **45** | **4** | **3** | **0** | **0** | **5** | **1** | **2** | **12** |
